# The Effect of Experimental *Fusarium* Mycotoxicosis on Microbiota Diversity in Porcine Ascending Colon Contents

**DOI:** 10.3390/toxins6072064

**Published:** 2014-07-14

**Authors:** Małgorzata Piotrowska, Katarzyna Śliżewska, Adriana Nowak, Łukasz Zielonka, Zofia Żakowska, Magdalena Gajęcka, Maciej Gajęcki

**Affiliations:** 1 Institute of Fermentation Technology and Microbiology, Lodz University of Technology, Wólczańska 171/173, Łódź 90-924, Poland; E-Mails: katarzyna.slizewska@p.lodz.pl (K.S.); adriana.nowak@p.lodz.pl (A.N.); zofia.zakowska@p.lodz.pl (Z.Z.); 2 Department of Veterinary Prevention and Feed Hygiene, University of Warmia and Mazury in Olsztyn, Oczapowskiego 13/19, Olsztyn 10-717, Poland; E-Mails: lukasz.zielonka@uwm.edu.pl (L.Z.); mgaja@uwm.edu.pl (M.Gajecka); gajecki@uwm.edu.pl (M.Gajecki)

**Keywords:** zearalenone, deoxynivalenol, pigs, microbiota, ascending colon, functional diversity, mycotoxicosis, low dosis

## Abstract

The objective of the study was to determine the effect of exposure of pigs to the *Fusarium* mycotoxins zearalenone (ZEN) and deoxynivalenol (DON), administered together and separately, on the colon microbiota. An experiment was conducted for 42 days on gilts, randomly assigned to four groups and administered either ZEN, DON, ZEN+DON, or a placebo. The number of aerobic mesophilic bacteria, yeasts, molds, anaerobic *Clostridium perfringens*, fecal streptococci, *Enterobacteriaceae*, *Escherichia coli*, and lactic acid bacteria (LAB) were determined in the contents of the ascending colon. The influence of mycotoxins on the functional diversity of the colonic microbiota was assessed using EcoPlate tests (Biolog). Analysis revealed the predominance of LAB in all groups of pigs. Zearalenone, administered separately and together with DON, was found to have an adverse effect on mesophilic aerobic bacteria, but only after long exposure to this mycotoxin. During the six weeks of the experiment, the concentration of *C. perfringens*, *E. coli*, and other bacteria in the family *Enterobacteriaceae* was most considerably reduced in the experimental groups exposed to zearalenone, both separately and together with DON. Mycotoxins also affected the functional biodiversity of microorganisms. Both Shannon’s diversity index and the number of catabolized substrates in Biolog plate (the R index) were much higher in the group subjected to mixed mycotoxicosis.

## 1. Introduction

Mycotoxins are secondary metabolites produced by various molds belonging mainly to *Aspergillus*, *Penicillium* or *Fusarium* genera. The worldwide problem of feedstuffs contamination with mycotoxins is important from the economic perspective and from the point of view of animal health [1]. Feedstuffs that are excessively contaminated with mycotoxins adversely affect the health of livestock, lower the absorption of nutrients, reduce weight gain, compromise immunity, and disturb reproduction, leading to body dysfunctions, greater susceptibility to diseases, and finally to suboptimal production performance [1,2]. Livestock animals exhibit different symptoms following exposure to mycotoxins in feedstuffs, while the degree and consequences of those symptoms depend on genetic factors (species, breed, individual differences), physiological factors (age, diet), and environmental factors (climate, livestock production methods) [2].

Recently, there has been an increased interest, particularly in European countries, in mycotoxins produced by fungi of the genus *Fusarium* [3]. A study of Wiśniewska and co-workers [4] showed that the species most widespread in Polish wheat are *Fusarium culmorum*, *F. graminearum*, and *F. avenaceum*. Grains affected by these fungi have been shown to contain considerable amounts of deoxynivalenol and zearalenone. These mycotoxins are also most often found in livestock feedstuffs. A study conducted in Europe and Asia in the years 2004–2011 showed that out of 17,000 samples of feed components and feedstuffs, 36% were contaminated with zearalenone (ZEN) (the average ZEN concentration in the positive samples was 0.28 mg/kg, and the maximum ZEN concentration was 26.7 mg/kg) and 55% were contaminated with deoxynivalenol (DON) (the average DON concentration in the positive samples was 0.96 mg/kg, and the maximum DON concentration was 50.3 mg/kg). More than 50% of the samples were found to contain at least two toxins [5]. The above average feed contamination levels exceed the limits specified by the EU Commission’s recommendations, according to which the concentration of DON and ZEN in feedstuffs for pigs may not exceed 0.9 mg/kg and 0.25 mg/kg, respectively, while for gilts and piglets there is an additional limit on ZEN amounting to 0.1 mg/kg [6].

Zearalenone is a macrocyclic lactone that is primarily generated, together with over 120 of its derivatives, by *Fusarium graminearum*, *F. culmorum*, *F. cerealis*, and *F. equiseti* both in the field and during the storage of corn, barley, sorghum, and soya in disadvantageous environmental conditions [3]. In animals, ZEN is transformed into α- and β-zearalenol, of which the former binds to estrogen receptors much more strongly than ZEN.

Exposure to ZEN leads to changes in the reproductive system of animals, such as edematous uterus, ovarian cysts, and increased follicular maturation [7,8,9]. The intensity of zearalenone mycotoxicosis in pigs depends on the dose. In gilts, administration of ZEN at relatively low doses (1.5 to 2 mg/kg in diet) during three to seven days leads to a swelling and thickening of the vaginal and vulvar wall, an increased uterus mass, atrophic ovaries, and intensified cell proliferation [10,11,12]. Long-term exposure to ZEN in the doses ranged of 1 to 5 mg/kg result in hyperestrogenism and increased pig mortality, while exposure to doses exceeding 100 mg/kg causes sow infertility [10,13,14].

Deoxynivalenol is a mycotoxin produced by *Fusarium culmorum*, *F. graminearum*, *F. crookwellense*, and *F. sambucinum*. Together with its derivatives 3-acetyl-DON and 15-acetyl-DON, this toxin is a widespread feed contaminant. Long-term exposure to DON may lead to anorexia, reduced weight gain, diminished nutritional efficiency, and immune modulation. DON causes protein biosynthesis inhibition and induces pro-inflammatory cytokine production [15,16,17]. Rotter and co-workers [18] demonstrated that pigs are the most sensitive animals to DON. Chronic exposure to DON in the range of 1–2 mg/kg of feed resulted in decreased appetite, while a dose of 3 mg/kg was the direct cause of reduced body temperature and changes in the gastric wall in piglets. Long-term exposure of pig to 4 mg of DON per kg of feed decrease feed intake, weight gain, and efficiency of feed utilization throughout the experiment with pigs [19]. Calculations from the literature data show that the growth rate of pigs was reduced by about 7% for each mg DON/kg increase in the diet. Symptoms of total refusal of feed intake occurred beyond 12 mg DON/kg of feed. DON in very high doses (0.1 to 0.3 mg/kg bw or 20 mg/kg of feed) causes vomiting in 9–15 kg piglets [20].

An appropriate composition of the intestinal microbiota of animals, as well as the quantitative and qualitative stability of that ecosystem, is an important factor in ensuring animal health [21]. Intestinal bacteria through immune responses and metabolic products are closely connected to the host. The gut microbiota provides nutritional and protective function to the animals, in the stimulation of host immunity, production of fermentation products, and prevention of colonization by the pathogens [21]. Apart from the benefits, microorganisms can negatively impacts the animals. The potential role of mycotoxins and intestinal microflora in inflammatory bowel diseases (IBD) was reviewed by Maresca and Fantini [22]. The intestinal microbiota is influenced by environmental conditions, the health of the animal, the individual characteristics of the animal, as well as by the type and quality of the fodder [23]. An inappropriate feeding process or biological/chemical contamination of the feed may result in changes in the intestinal ecosystem that cause excessive growth of pathogenic microorganisms and a reduction in the numbers of beneficial bacteria, leading to diseases and losses in livestock production. Pathogenic microorganisms generate toxic metabolites and so-called fecal enzymes that may give rise to carcinogenic substances or transform procarcinogenic compounds into carcinogenic ones [24]. The data available regarding interaction of mycotoxins with bacteria mainly concern the ability of intestinal microbiota to detoxify mycotoxins. It was demonstrated that microbiota of the large intestine of chickens (CLIC) and rumen fluid transformed pure DON to deepoxy DON [25,26]. No alteration of the toxin during incubation with bacteria from large intestines of swine was observed [25]. The complete transformation of DON to dE-DON by microorganisms from fish digesta were obtained by Guan and co-workers [27] The role of microorganisms in detoxification of mycotoxins was reviewed by Kabak and Dobson [28,29].

Studies concerning the effect of DON and ZEN on the pig intestinal microbiota communities are limited. *In vitro* studies of Ali-Vehmas and co-workers [30] showed that DON did not influenced on *Staphylococcus aureus*, *Escherichia coli*, and *Yersinia enterocolitica* growth yield. Inhibition of *Streptococcus agalactiae* was observed only. Wache and co-workers [31] demonstrated that DON at a dose of 2.8 mg/kg of feed increased the amount of intestinal aerobic bacteria and decrease of anaerobic sulfite-reducing bacteria. In the study of Saint-Cyr [32] a significant decrease of *Escherichia coli* amount in feces of rats exposed to DON at 100 μg/kg bw after 27 days was observed. The mechanism by which this mycotoxin is able to cause certain quantitative changes in bacterial gut flora is not known.

Changes in the quantitative and qualitative composition of the intestinal microbiota caused by dietary factors, including mycotoxins, may be studied using culture methods, but not all microorganisms can be detected on microbiological media. Thus, alternative molecular and biochemical methods may be used for this purpose. The microbial community structure may be investigated by means of terminal restriction fragment length polymorphism (T-RFLP) analysis targeting the 16S ribosomal DNA [33,34]. Another technique apply to evaluation of the diversity of microorganisms in biocenosis is community-level physiological profiling (CLPP) using EcoPlates (Biolog) system. This method based on analysis of the utilization pattern of individual carbon substrates by microbial community. This method is fast and convenient. Community structure can be estimated by calculating Shannon’s diversity index (*H*) and richness value (*R*), *i.e.*, the number of catabolized C substrates [35,36]. In the literature, there are only a few reports concerning the use of this method for studying the intestinal microbiota in animals [37,38].

The objective of the present study was to determine the effect of the *Fusarium* mycotoxins ZEN and DON administered together and separately at NOAEL (no observable adverse effect level) doses on the colonic microbiota of sexually immature gilts. The doses of 40 μg ZEN/kg bw/day and 12 μg DON/kg bw/day were used [13,17].

## 2. Results and Discussion

During the experiment, no bacteria of the genus *Salmonella* were detected in any colonic contents sample, which means that the gilts were in good health and the feed was of good quality. Table 1, Table 2 and Table 3 show changes in the amounts of aerobic mesophilic bacteria (AMB), yeasts and molds (Y&M), and lactic acid bacteria (LAB) in the contents of the ascending colon. The results that were found to be outliers in Grubbs’ test were excluded from mean calculation. Analysis of raw data shows that in some cases there were considerable discrepancies between samples from a given group of gilts in a given week, which indicates the presence of individual differences between the studied animals.

Amounts of particular groups of microorganisms revealed the predominance of LAB in all experimental groups. The LAB amounts remained above 10^9^ CFU/g throughout the experiment (five weeks) and no statistically significant differences were found between the studied groups of gilts. Only on week six a significant differences between DON and CONTROL group (*p* < 0.05) were observed. The high levels of LAB are consistent with the literature data according to which in the small intestine of healthy animals LAB are the predominant bacteria [39]. The large amount of LAB in the digestive tract is linked to the age of the gilts, and it may be maintained at this level by appropriate feeding, including probiotic supplementation. LAB play a beneficial role for the host, producing substances such as short-chain fatty acids (acetic, butyric, propionic), amino acids, and B vitamins, as well as other antimicrobial metabolites, such as bacteriocins, which prevent colonization of the digestive tract by pathogens and stimulate the immune system of the host [21,39,40,41]. Furthermore, LAB can bind mycotoxins, including *Fusarium* toxins, from their environment [42,43]. The capacity to remove of above 60% of DON initial concentration (1.5 µg/mL) from model liquid media by viable and heat-inactivated cells of *Lactobacillus* species was reported [43]. According to El-Nezami and co-workers [44] this phenomenon occurs as a result of mycotoxin adsorption to bacterial cell wall.

**Table 1:** The influence of mycotoxins on the amounts (log CFU/g) of lactic acid bacteria (LAB) in the contents of the ascending colon.

Weeks of experiment	Experimental Groups			
	DON	ZEN	ZEN+DON	CONTROL
0	8.95 ^a,A^ ± 0.36	8.95 ^a,A^ ± 0.36	8.95 ^a,A^ ± 0.36	8.95 ^a,A^ ± 0.36
1	9.32 ^a,A^ ± 0.14	9.28 ^a,A^ ± 0.21	9.24 ^a,A^ ± 0.05	8.64 ^a,A^ ± 0.62
2	9.20 ^a,A^ ± 0.03	9.16 ^a,A^ ± 0.20	8.79 ^a,A^ ± 0.35	8.87 ^a,A^ ± 0.72
3	9.27 ^a,A^ ± 0.15	9.07 ^a,A^ ± 0.25	9.12 ^a,A^ ± 0.29	9.21 ^a,A^ ± 0.16
4	8.69 ^a,A^ ± 0.33	8.69 ^a,A^ ± 0.43	8.95 ^a,A^ ± 0.20	9.26 ^a,A^ ± 0.21
5	8.82 ^a,A^ ± 0.20	8.80 ^a,A^ ± 0.35	8.61 ^a,A^ ± 0.45	9.02 ^a,A^ ± 0.21
6	8.98 ^a,B^ ± 0.06	8.72 ^a,A^ ± 0.16	8.50 ^a,A^ ± 0.30	9.00 ^a,AB^ ± 0.48

Note: The values represent means from three animals in each week ± SD. The different lowercase (a) letters for average values in columns indicate statistically significant differences in the concentration of particular microorganisms within the time of sampling (*p* < 0.05). The different capital letters (A,B) for average values in rows indicate statistically significant differences in the concentration of particular microorganisms within the group of sampling in each week (*p* < 0.05).

**Table 2:** Mycotoxin-induced changes in the amounts (log CFU/g) of aerobic mesophilic bacteria (AMB) in the contents of the ascending colon.

Weeks of experiment	Experimental Groups			
	DON	ZEN	ZEN+DON	CONTROL
0	7.79 ^a,A^ ± 1.77	7.79 ^a,A^ ± 1.77	7.79 ^a,A^ ± 1.77	7.79 ^a,A^ ± 1.77
1	6.21 ^a,AB^ ± 1.03	6.12 ^b,A^ ± 0.18	5.98 ^ac,A^ ± 0.96	7.66 ^a,B^ ± 0.64
2	5.43 ^a,A^ ± 0.34	5.71 ^b,A^ ± 0.34	6.48 ^ac,A^ ± 1.44	5.57 ^a,A^ ± 0.73
3	5.91 ^a,A^ ± 0.83	5.90 ^b,A^ ± 0.23	6.38 ^ac,A^ ± 0.84	5.52 ^a,A^ ± 0.49
4	5.17 ^a,A^ ± 0.99	5.79 ^b,A^ ± 0.54	5.48 ^c,A^ ± 0.11	5.54 ^a,A^ ± 1.00
5	6.30 ^a,A^ ± 1.11	5.82 ^b,A^ ± 0.29	6.13 ^ac,A^ ± 1.99	5.44 ^a,A^ ± 0.64
6	6.06 ^a,A^ ± 0.88	4.79 ^c,B^ ± 0.22	5.59 ^c,A^ ± 0.17	5.55 ^a,AB^ ± 1.10

Note: The values represent means from three animals in each week ± SD. The different lowercase letters (a–c) for average values in columns indicate statistically significant differences in the concentration of particular microorganisms within the time of sampling (*p* < 0.05). The different capital letters (A,B) for average values in rows indicate statistically significant differences in the concentration of particular microorganisms within the group of sampling in each week (*p* < 0.05).

The mean initial amount of aerobic mesophilic bacteria (AMB) in the contents of the ascending colon was 7.79 log CFU/g, and remained at a similar level in all groups of gilts throughout the experiment. Only in the ZEN group, a statistically significant (*p* < 0.05) decrease in the AMB count by 1 or 2 log units as compared to *t* = 0 was observed in week one. In week six, in the ZEN and ZEN+DON groups a significant reduction in AMB amounts was observed in comparison to the preceding weeks (Table 2). These results show an adverse effect of ZEN, whether administered separately or with DON, on AMB, following prolonged exposure to the mycotoxin. A study by Burel and co-workers [45], concerning another *Fusarium* mycotoxin, fumonisin B1, indicates that low doses of this mycotoxin did not affect the porcine microbiota. Over the consecutive weeks of the experiment, the bacterial amounts in all experimental groups were slightly lower than those reported in the literature data. According to Drew and co-workers [46], depending on the type of grain in the feed, the amount of aerobic bacteria is in the range from 6.56 log CFU/g to 7.15 log CFU/g in the ileum and 7.74 log CFU/g on average in the cecum. We found no effect of DON on AMB concentration during the experiment. The opposite results were obtained in studies of Wache and co-workers [31], in which the amount of aerobic mesophilic bacteria in swine fecal samples increased by more than 2 log in the first week of exposure to DON with a dose of 2.8 mg/kg in feed.

During the experiment, fungal amounts in the colonic contents varied within 1 log unit above or below the initial level, that is, 4 log CFU/g, but these differences were not statistically significant. Only in the ZEN group, the concentration of fungi decreased significantly to 3.29 log CFU/g (Table 3). The most numerous microorganisms in this category were the mold *Geotrichum candidum* and the yeast *Candida glabrata*. No correlation was found between experimental groups and the predominant species of fungi.

**Table 3:** Mycotoxin-induced changes in the amounts (log CFU/g) of yeasts and molds (Y&M) in the contents of the ascending colon.

Weeks of experiment	Experimental Groups			
	DON	ZEN	ZEN+DON	CONTROL
0	4.04 ^a,A^ ± 0.09	4.04 ^a,A^ ± 0.09	4.04 ^a,A^ ± 0.09	4.04 ^a,A^ ± 0.09
1	4.01 ^a,A^ ± 0.16	3.86 ^a,A^± 0.19	4.19 ^a,AB^ ± 0.46	5.58 ^a,B^ ± 0.94
2	3.33 ^a,A^ ± 0.30	3.57 ^b,AB^ ± 0.29	4.81 ^a,B^ ± 1.06	4.16 ^a,AB^ ± 1.06
3	3.77 ^a,AB^ ± 0.87	3.93 ^a,AB^ ± 0.68	4.99 ^a,A^ ± 0.49	3.85 ^a,B^ ± 0.19
4	4.13 ^a,A^ ± 1.03	4.90 ^a,A^ ± 0.96	3.79 ^a,A^ ± 0.56	4.18 ^a,A^ ± 0.75
5	4.49 ^a,A^ ± 0.67	3.50 ^a,A^ ± 0.54	4.33 ^a,A^ ± 1.71	3.89 ^a,A^ ± 0.34
6	4.67 ^a,B^ ± 0.40	3.29 ^b,A^ ± 0.37	3.92 ^a,AB^ ± 0.47	3.74 ^a,AB^ ± 0.84

Note: The values represent means from three animals in each week ± SD. The different lowercase letters (a,b) for average values in columns indicate statistically significant differences in the concentration of particular microorganisms within the time of sampling (*p* < 0.05). The different capital letters (A,B) for average values in rows indicate statistically significant differences in the concentration of particular microorganisms within the group of sampling in each week (*p* < 0.05).

Analysis of the microbiota in the studied samples showed that the most abundant species were Gram-negative oxidase-negative bacteria belonging to the family *Enterobacteriaceae*, such as *Pantoea agglomerans*, *Enterobacter cloacae*, and *Enterobacter aerogenes*, and also Gram-positive cocci (*Micrococcus luteus* and *Staphylococcus xylosus*). No significant changes in the composition of the microbiota were found over the course of the experiment or between the experimental groups.

**Figure 1:**
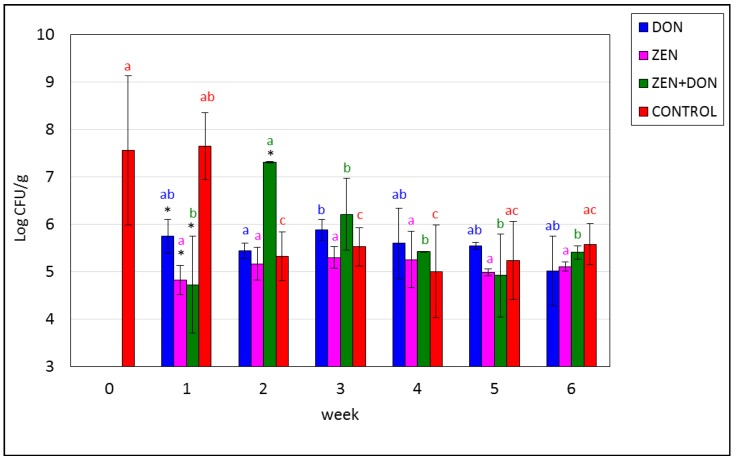
Mycotoxin-induced changes in the amounts of *Enterobacteriaceae* family bacteria in the ascending colon. The values plotted in the charts represent means from three samples in each week ± SD. Results significantly different from control in each week (*p* < 0.05) are marked by color asterisks. The different color letters for average values in each group indicate statistically significant differences in the concentration of particular microorganisms within the time of sampling (*p* < 0.05).

**Figure 2:**
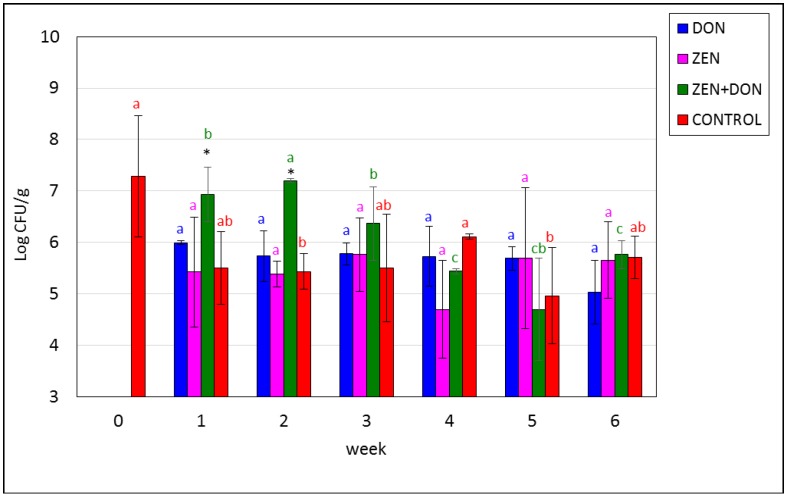
Mycotoxin-induced changes in the amounts of *Escherichia coli.* The values plotted in the charts represent means from three samples in each week ± SD. Results significantly different from control in each week (*p* < 0.05) are marked by black asterisks. The different color letters for average values in each group indicate statistically significant differences in the concentration of particular microorganisms within the time of sampling (*p* < 0.05).

**Figure 3:**
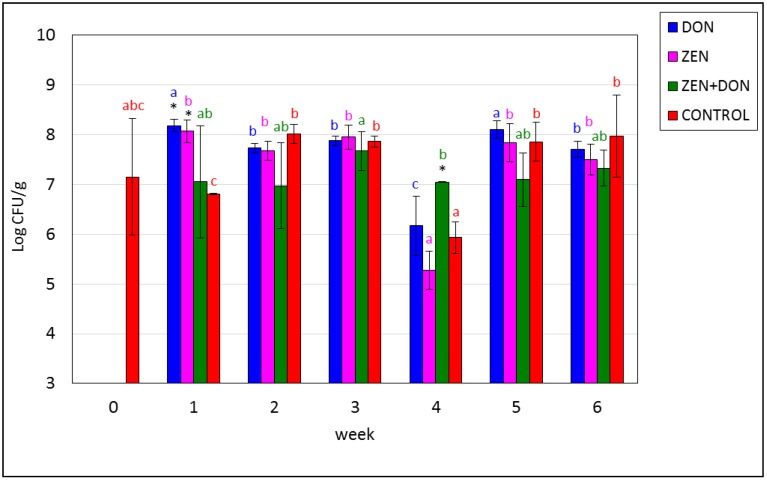
Mycotoxin-induced changes in the amounts of fecal enterococci in the ascending colon. The values plotted in the charts represent means from three samples in each week ± SD. Results significantly different between weeks in each group (*p* < 0.05) are marked by color asterisks. The different color letters for average values in each group indicate statistically significant differences in the concentration of particular microorganisms within the time of sampling (*p* < 0.05).

**Figure 4:**
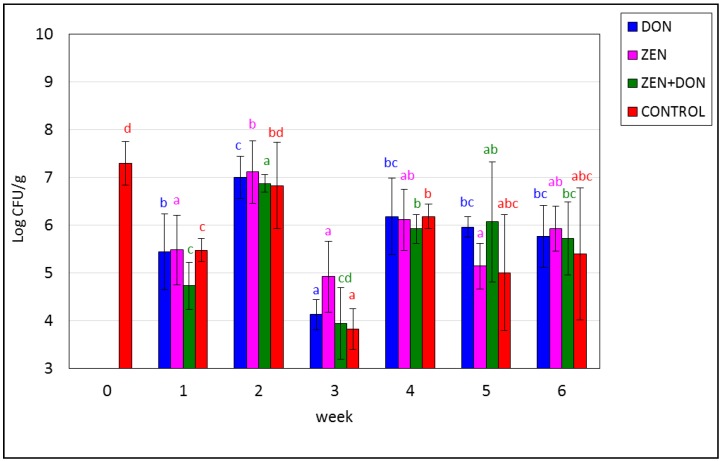
Mycotoxin-induced changes in the amounts of *Clostridium perfringens* in the ascending colon. The values plotted in the charts represent means from three samples in each week ± SD. Results significantly different from control in each week (*p* < 0.05) are marked by black asterisks. The different color letters for average values in each group indicate statistically significant differences in the concentration of particular microorganisms within the time of sampling (*p* < 0.05).

Figure 1, Figure 2, Figure 3 and Figure 4 show the dynamics of changes in the amounts of fecal bacteria throughout the experiment. The first test examined bacteria belonging to the family *Enterobacteriaceae*. Their initial average amount in the contents of the ascending colon was 7.82 log CFU/g (6.6 × 10^7^ CFU/g). This concentration decreased in the ZEN and ZEN+DON groups by approx. 2 log units in week one and remained stable until the end of the experiment. In the other experimental groups, concentration of these bacteria from week two varied slightly, but not statistically significantly (*p* > 0.05). Differences at *p* < 0.05 were reported: in the ZEN+DON group between week two and the following ones and in the CONTROL group between week one and the remaining (Figure 1).

The next test determined the concentration of *Escherichia coli*, which also belong to the family *Enterobacteriaceae* and are part of the normal microbiota of the animal digestive tract. The maximal initial amount of these bacteria in the contents of the ascending colon was 8.38 log CFU/g (average 7.3 log CFU/g). Their concentration fluctuated slightly during the experiment, but the differences were not statistically significant, except for the ZEN+DON group, where the concentration of *E. coli* gradually decreased beginning in week three, to achieve a minimum in week five at 4–5 log CFU/g. Differences at *p* < 0.05 were reported: in ZEN+DON group between week two and the next, and in the CONTROL group between week 0 and weeks two and five. Differences (*p* < 0.05) in weeks one and two, between ZEN+DON group and the CONTROL group, were noted (Figure 2). There are no differences (*p* > 0.05) between groups exposed on single mycotoxins and the CONTROL group. These results are opposite to research Saint-Cyr and co-workers [32], which found the decreasing of *E. coli* level after 27 days of rats exposition on DON at dose 100 µg/kg bw.

The predominance of fecal bacteria belonging to the family *Enterobacteriaceae* is a natural phenomenon in the colon. These bacteria may exhibit the activity of the so-called fecal enzymes β-d-glucosidase and β-d-glucuronidase. The excessive activity of these enzymes may give rise to mutagenic, carcinogenic, and genotoxic products, which may promote colon cancer [47]. However, the presence of high concentrations of LAB may counteract those effects [48]. The ratio of the amount of LAB to *E. coli* was approximately 2, which is a positive finding, indicating a correct composition of the microbiota [39].

The amount of fecal streptococci remained stable throughout the experiment and amounted to approx. Eight log CFU/g of colonic contents, except for the ZEN group, where it dramatically declined and increased in weeks three and four (*p* < 0.05). Differences at *p* < 0.05 between DON group in one week and two, three, four and six weeks and in CONTROL group between 1 and the remaining dates were also observed (Figure 3).

The initial amount of the spore-forming sulfite-reducing bacteria *Clostridium perfringens* (CP) amounted to approx. Seven log CFU/g. In all experimental groups, the concentration of these bacteria fluctuated along the first four weeks, and subsequently reached a stable level. ZEN was not found to adversely affect the concentration of CP. In week three, there was a pronounced decline in the concentration of these bacteria, even by 2 or 3 log units (*p* < 0.05) in the experimental groups administered DON, separately and with ZEN, as well as in the control group. A similar lack of differences between the control group and a group exposed to DON was found by Wache and co-workers [31]. In their study, the concentration of these bacteria in pig feces was higher than in the study presented herein (over 7 log CFU/g). Anaerobic bacteria of the genus *Clostridium* are the second most abundant bacteria (after LAB) in the porcine colon. These bacteria appear in the intestines following piglet weaning and the shift from milk to fodder, and may actually become predominant in the small intestine [46]. Gram-positive anaerobic bacteria, including LAB, are predominant in the animal digestive tract, with the number of their species reaching 500 in some cases. In the colon, the concentration of these bacteria may amount to as much as 10^10^–10^12^ CFU/g [21].

**Figure 5:**
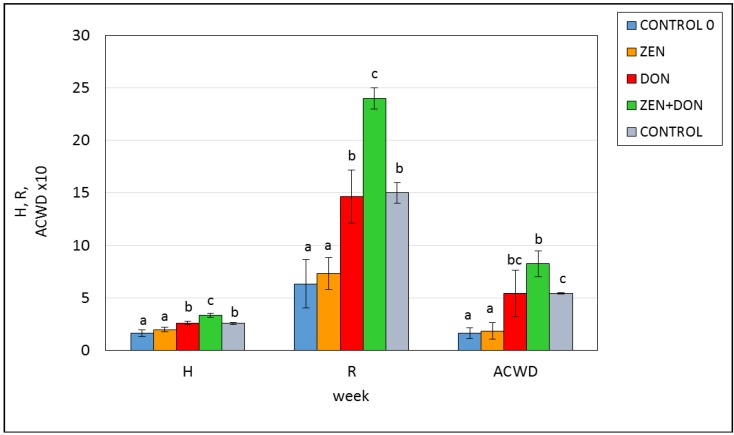
The influence of mycotoxins on the functional diversity of the microbiota in the ascending colon. *H*—Shannon’s index; AWCD—average well color development; *R*—richness index; CONTROL 0—control sample before exposition; ZEN, DON, ZEN+DON; CONTROL—samples after six weeks of exposition by ZEN, DON, ZEN+DON, and *placebo*, respectively; the different letters for average values indicate statistically significant differences in the *H*, *R*, or ACWD value within the group (*p* < 0.05).

The composition and metabolic activity of the microbiota in the digestive tract is affected by many factors, including the diet, and especially the presence of toxic substances, which may inhibit or stimulate the enzymatic activity of microorganisms. Analysis of the results obtained using the Biolog system revealed low initial metabolic activity of the microbiota of the ascending colon. Shannon’s index amounted to 1.65, and the number of catabolized substrates (the *R* index) was 5.33 on average (Figure 5). In week six of the experiment, when the porcine microbiota was quantitatively rather stable, the control group and the groups exposed to DON alone and with ZEN exhibited higher *H* and *R* values than at the beginning of the experiment (*p* < 0.05), which indicates higher intensity of substrate use, and higher functional diversity of the microorganisms. The highest *H* value (3.36) was found in the ZEN+DON experimental group. The colonic microbiota of this group metabolized 24 out of 31 substrates. This means that simultaneous exposure to both toxins leads to intensified metabolic activity in the intestinal microbiota.

**Figure 6:**
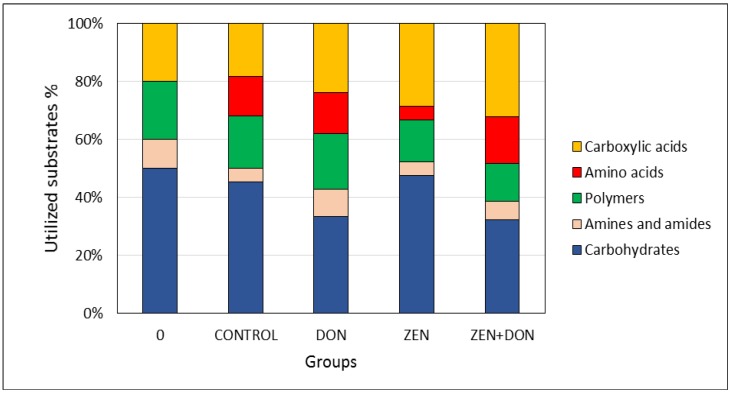
The influence of mycotoxins on the consumption of EcoPlate substrates by the microbial community of the ascending colon.

At the beginning of the experiment, the substrates metabolized to the greatest degree among the studied options were carbohydrates, which accounted for 50% of the total number of metabolized substrates. At *t* = 0, the colonic microbiota of the control group was not found to metabolize amino acids (Figure 6).

After six weeks of exposure to mycotoxins, the share of carbohydrates in total metabolized substrates fell to 35% in the DON and ZEN+DON groups, while in the other experimental groups no changes in this category of substrates were detected. The microorganisms isolated from the ZEN+DON and ZEN groups exhibited increased metabolism of carboxylic acids, which accounted for 30% of the total pool of metabolized substrates. In these groups, as well as in the control group, microorganisms revealed higher activity in terms of metabolizing amino acids. Such activity may lead to the formation of toxic metabolites, such as ammonia, amines, phenolics, and indoles, which adversely affect intestinal cells, increase gut epithelial cell turnover, elevate intestinal peristalsis, and diarrhea in pigs, and, thus, hamper growth performance [21].

The results concerning the number and biodiversity of microorganisms, as well as those concerning their metabolic activity, show that these parameters are affected to the greatest extent by exposure to a mixture of the mycotoxins ZEN and DON. According to the literature data, low doses of *Fusarium* mycotoxins, including ZEN, which do not result in readily observable symptoms in the animals exposed to them, may, nevertheless, lead to changes at the cellular level [49].

## 3. Experimental Section

### 3.1. In Vivo Experiment

All experimental procedures involving animals were conducted according to the Polish legal regulations concerning experiments on animals (following a decision issued by the Local Ethical Committee for Experiments on Animals No. 88/N of 16 December 2009).

The experiment was conducted at the Department of Veterinary Prevention and Feed Hygiene, Faculty of Veterinary Medicine, University of Warmia and Mazury in Olsztyn, Poland. The procedures involved 75 clinically healthy gilts with an initial body weight of approx. 25 ± 2 kg. The gilts were divided into groups and penned with *ad libitum* access to water. The animals were randomly assigned to three experimental groups (ZEN, DON, and ZEN+DON), with 18 gilts in each, and to one control group (CONTROL) with 21 gilts. Each day, animals in the experimental groups were administered *per os* 40 μg of ZEN/kg bw (body weight), 12 μg of DON/kg bw, or 40 μg ZEN/kg bw + 12 μg DON/kg bw. The animals were weighed every seven days. Amounts of toxin application were dependent on the body weight and updated weekly. Analytical samples of the studied mycotoxins were administered *per os* daily in gelatin capsules before the morning feeding. A placebo was administered in the control group. In all experimental groups, the mycotoxins were administered at NOAEL (no observable adverse effect level) doses [50,51,52]. The experiment was conducted over 42 days. Gilts were sacrificed on a weekly basis. Each week, 3 gilts from each experimental group were sacrificed (12 gilts each time), except for week 0, when only 3 gilts from the control group were sacrificed. Each time, the contents of the ascending colon were collected from 3 gilts from each experimental group.

The study material consisted of samples of contents from the ascending colon collected in a sterile manner and delivered to the microbiological laboratory under refrigerated conditions. The samples were collected at the beginning of the experiment (week 0) and in subsequent weeks (week 1, 2, 3, 4, 5, and 6).

### 3.2. Toxins

Deoxynivalenol and zearalenone were synthesized and standardized at the laboratory of the Department of Chemistry, Poznań University of Life Sciences by Prof. Piotr Goliński team.

Biosynthesis of DON and ZEN was conducted by *Fusarium graminearum* and *F. culmorum* strains on rice grain by 4 weeks in 25 °C with regular shaking. The methods of extraction, purification and analysis of ZEN was presented in [53]. In order to obtain pure DON the dried and milled seeds was extracted by methanol:water (3:1) and defatted with heptane. The liquid-liquid extraction with ethyl acetate was conducted. Obtained extract was evaporated do dryness and dissolved in chloroform. Extract was evaporated again and dissolved in ethyl acetate. In the next step the extract was applied on the column filled with activated carbon and silica gel. DON was eluted by ethyl acetate and their purity in each fraction determined by HPLC method [4].

Mycotoxin samples were diluted in 300 μL of 96% ethyl alcohol (POCH, Gliwice, Poland) to obtain the required doses. The resulting solutions were stored at room temperature for 12 h to evaporate the solvent.

### 3.3. Microbiological Analysis

Samples of colonic contents were analyzed microbiologically by the culture method using media according to relevant ISO standards (4833:2004, 21727-1:2008, 7937:2005, 7899-2:2004, 21528-2:2005, 16649-2:2004, 6579:2003). Aerobic mesophilic bacteria (AMB) were determined on Plate Count Agar (PCA) at 30 °C, yeasts and molds (Y&M) on Rose Bengal Chloramphenicol Agar (RBC) at 25 °C, sulfite-reducing anaerobic bacteria *Clostridium perfringens* (CP) on Tryptose Sulfite Cycloserine Agar (TSC) at 37 °C under anaerobic conditions, fecal streptococci (SF) on Bile Aesculin Agar at 37 °C, members of the family *Enterobacteriaceae* (EE) on Violet Red Bile Dextrose Agar (VRBD) according to Mossel at 37 °C, *Escherichia coli* (EC) on Chromocult^®^ TBX Agar (TBX) at 44 °C, lactic acid bacteria (LAB) on *Lactobacillus* Agar according to De Man, Rogosa, and Sharpe (MRS) [54]. Furthermore, 1 g colonic contents samples were tested for the presence of *Salmonella*. The following steps were carried out: pre-enrichment in buffered peptone water (37 °C, 18 h), selective enrichment in Muller-Kauffmann Tetrathionate-Novobiocin Broth (MKTTn) (37 °C, 24 h) and in Rappaport-Vassiliadis Broth (RVS) (41.5 °C, 24 h), and selective differentiation on Xylose Lysine Deoxycholate Agar (XLD) and on Brilliant Green Agar (BGA) (37 °C, 24 h). All media were from Merck GmbH (Darmstadt, Germany).

### 3.4. Microorganisms Identification

The isolated pure cultures of bacteria and fungi were transferred to TSA and Czapek-Dox medium (Merck). Bacteria were identified using the standard methods based on morphological features, Gram staining, and the presence of oxidase (Bactident^®^ Oxidase, Merck GmbH, Darmstadt, Germany), as well as by biochemical methods using API tests (BioMerieux, Lyon, France). Yeasts were identified using API tests (BioMerieux, Lyon, France). Molds were identified based on their macroscopic and microscopic characteristics according to identification keys [55,56]. Identification was carried out for the prevalent microflora.

### 3.5. Determination of the Functional Diversity of the Intestinal Microbiota

Biolog EcoPlates (Biolog, Hayward, CA, USA) consist of 96 wells containing 31 different sources of carbon and water as a control, in three replicates. The carbon sources are grouped, according to Weber and Legge [57], into carbohydrates, polymers, carboxylic and acetic acids, amino acids, and amines/amides (Table 4). The tests were conducted on the colonic contents at the beginning of the experiment and in week 6, for each group of gilts. Sterile deionized water (12 mL) was shaken with 1 g of colonic contents for 20 min at 20 °C. Each well of the plate was inoculated with 150 µL of sample suspension and incubated at 30 °C. Optical density (OD) was measured every 24 h until 120 h with an ASYS UVM340 microtiter spectrophotometer at 590 nm.

**Table 4:** Biolog Ecoplate carbon source guild grouping [57].

ID	Substrate	Group
C0	Water (blank)	-
C1	Pyruvic acid methyl ester	Carbohydrate
C2	Tween 40	Polymers
C3	Tween 80	Polymers
C4	α-Cyclodextrin	Polymers
C5	Glycogen	Polymers
C6	d-Cellobiose	Carbohydrate
C7	a-d-Lactose	Carbohydrate
C8	b-Methyl-d-glucoside	Carbohydrate
C9	d-Xylose	Carbohydrate
C10	i-Erythritol	Carbohydrate
C11	d-Mannitol	Carbohydrate
C12	*N*-Acetyl-d-glucosamine	Carbohydrate
C13	d-Glucosaminic acid	Carboxylic & acetic acids
C14	Glucose-1-phosphate	Carbohydrate
C15	d,l-a-Glycerol phosphate	Carbohydrate
C16	d-Galactonic acid γ–lactone	Carboxylic & acetic acids
C17	d-Galacturonic acid	Carboxylic & acetic acids
C18	2-Hydroxybenzoic acid	Carboxylic & acetic acids
C19	4-Hydroxybenzoic acid	Carboxylic & acetic acids
C20	c-Hydroxybutyric acid	Carboxylic & acetic acids
C21	Itaconic acid	Carboxylic & acetic acids
C22	a-Ketobutyric acid	Carboxylic & acetic acids
C23	d-Malic acid s	Carboxylic & acetic acids
C24	l-Arginine	Amino acids
C25	l-Asparagine	Amino acids
C26	l-Phenylalanine	Amino acids
C27	l-Serine	Amino acids
C28	l-Threonine	Amino acids
C29	Glycyl-l-glutamic acid	Amino acids
C30	Phenylethylamine	Amines/amides
C31	Putrescine	Amines/amides

Data were normalized by average well color development (AWCD), which was calculated as follows [58]:

AWCD = ƩOD_i_/31

where OD_i_ is the optical density value from each well corrected for the blank well values.

The threshold for a positive test is defined as a value higher than 0.25 after background correction. Richness (*R*) is defined as the total number of metabolized carbon source substrates. The Shannon-Weaver index (*H*) was calculated as follows [58]:

H = −Ʃp_i_ (lnp_i_)

where p_i_ is the ratio of the activity on each substrate (OD_i_) to the sum of activities on all substrates ƩOD_i_.

### 3.6. Statistical Analysis

In each week of the experiment and for each group of gilts, analysis was conducted in triplicate. Outliers were evaluated and rejected using Grubbs’ test. Arithmetic means and standard deviations were calculated. Differences between means were tested by variance analysis (one-way ANOVA) with the *post hoc* Tukey test. Probability (*p*) values of <0.05 were considered significant. Microcal ORIGIN v. 6.0 (Northampton, MA, USA) and Statistica v.10.0 (Stat Soft. Inc., Tulsa, OK, USA) were used for calculations.

## 4. Conclusions

The obtained results demonstrated that exposures of gilts to ZEA and DON in mixture at NOAEL concentration affects the concentration, biodiversity, and metabolic activity of the biocenosis of the porcine ascending colon. ZEA administered separately and with DON has an adverse effect on mesophilic aerobic bacteria 42 days of exposure. After the sixth week of the experiment, the amounts of *Clostridium perfringens*, *E. coli*, and other bacteria in the family *Enterobacteriaceae* were reduced. The simultaneous administration of ZEN and DON led to increased biodiversity of microorganisms and influenced the direction of their metabolism. The observed changes in the metabolic profile of the microbiota involve increased amino acid metabolism in the ZEN+DON group, which may be detrimental due to the formation of biogenic amines and procarcinogenic compounds. The results show that the exposure of gilts to the ZEN and DON administered simultaneously at NOAEL doses adversely affects the stability of the biocenosis of the porcine digestive tract, which is an important health indicator in animals. Future studies are needed to explain observed changes. Moreover, it is necessary to consider the officially recommended contamination limits for feedstuffs not only for individual mycotoxins, but also for their combinations.
